# Electrical Synapses Contribute to Sleep‐Dependent Declarative Memory Retention

**DOI:** 10.1111/ejn.70401

**Published:** 2026-01-23

**Authors:** Gordon B. Feld, Niels Niethard, Jianfeng Liu, Sandra Gebhardt, Lisa Kleist, Kerstin Brugger, Andreas Fritsche, Jan Born, Hong‐Viet V. Ngo, Manfred Hallschmid

**Affiliations:** ^1^ Clinical Psychology, Central Institute of Mental Health, Medical Faculty Mannheim University of Heidelberg Mannheim Germany; ^2^ Psychiatry and Psychotherapy, Central Institute of Mental Health, Medical Faculty Mannheim University of Heidelberg Mannheim Germany; ^3^ German Center for Mental Health (DZPG) Tübingen Germany; ^4^ Department of Psychology Heidelberg University Heidelberg Germany; ^5^ Institute of Medical Psychology and Behavioural Neurobiology University of Tübingen Tübingen Germany; ^6^ Wissenschaftskolleg zu Berlin Berlin Germany; ^7^ Department of Cognitive Sciences University of California, Irvine Irvine California USA; ^8^ German Center for Diabetes Research (DZD) Tübingen Germany; ^9^ Department of Internal Medicine, Division of Endocrinology, Diabetology, Angiology, Nephrology and Clinical Chemistry University Hospital of Tübingen Tübingen Germany; ^10^ Institute for Diabetes Research and Metabolic Diseases of the Helmholtz Center Munich at the University of Tübingen (IDM) Tübingen Germany; ^11^ Centre for Integrative Neuroscience University of Tübingen Tübingen Germany; ^12^ Department of Psychology University of Essex Colchester UK

**Keywords:** electrical synapse/gap junction, memory, sleep, systems memory consolidation

## Abstract

Sleep supports memory formation by neurophysiological mechanisms that are yet to be fully uncovered. We investigated the contribution of the direct coupling of neurons via electrical synapses (gap junctions). The administration of mefloquine (250 mg p.o. vs. placebo), an antimalarial, which blocks electrical synapses, to healthy young men before nocturnal sleep impaired the retention of word pairs learned before drug administration and disrupted the coupling of sleep spindles to EEG slow oscillations. In control experiments, in which participants received mefloquine before a consolidation interval of nocturnal wakefulness or after rather than before sleep, word‐pair memory retention was not affected by the drug, suggesting that electrical synapses specifically support the sleep‐dependent retention of verbal declarative memory. Irrespective of sleep, mefloquine enhanced the retention of sensorimotor memory assessed with a finger sequence tapping task. In supplemental experiments in rats, mefloquine administered i.p. at escalating doses of 20 and 40 mg/kg did not alter hippocampal sharp‐wave/ripple activity, a prominent mechanism of hippocampal memory replay. While mefloquine effects beyond gap junctions in the present experiments cannot be fully excluded, we conclude that electrical coupling enhances the oscillatory coordination between sleep spindles and slow oscillations and, thereby, supports systems memory consolidation.

## Graphical abstract

Blocking electrical synapses (gap junctions) in healthy humans by mefloquine impaired the sleep‐dependent retention of verbal declarative memory and improved sensorimotor memory consolidation irrespective of sleep. Mefloquine also disrupted the coupling of sleep spindles to EEG slow oscillations in humans but did not affect hippocampal sharp‐wave/ripple activity in rodents. These results indicate a role for electrical synapses in the systems consolidation of memory contents.

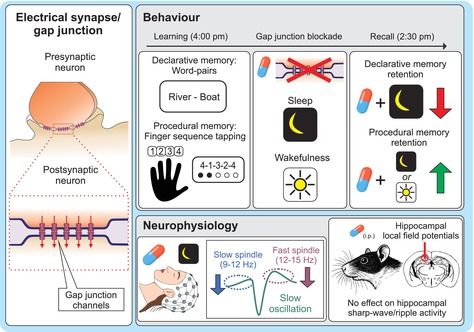

**Abbreviations**
Ag‐AgCl: silver‐silver chloride
AMPA: α‐amino‐3‐hydroxy‐5‐methyl‐4‐isoxazolepropionic acid
ANOVA: analysis of variance
CA1: hippocampal subfield 1
CA3: hippocampal subfield 3
CA4: hippocampal subfield 4
CV: coefficient of variation
Cx36: connexin 36
Cx50: connexin 50
DMSO: dimethyl sulfoxide
ECG: electrocardiogram
EEG: electroencephalogram
EOG: electrooculogram
EMG: electromyogram
GMP: good manufacturing practice
IC_50_
: half maximal inhibitory concentration
LFP: local field potential
NMDA: N‐methyl‐D‐aspartate
NREM: nonrapid eye movement
PVT: psychomotor vigilance task
REM: rapid eye movement
SD: standard deviation
SSS: Stanford Sleepiness Scale
SWS: slow‐wave sleep

## 1 Introduction

Sleep is an efficient memory enhancer (Stickgold 2005; Feld and Born 2017; Klinzing et al. 2019). During sleep, memory representations that were acquired during wakefulness are reactivated and replayed in a process that stabilizes and consolidates the initially labile traces into lasting memories (Rasch et al. 2007; Dupret et al. 2010; Yang et al. 2014). Memory replay is compressed in time by a factor of 5–10 (Ji and Wilson 2007), which greatly enhances the amount of information that can be processed by the sleeping brain. Replay occurs mainly during hippocampal sharp‐wave/ripple events (Diba and Buzsáki 2007). Consequently, suppressing these events electrically or optogenetically impairs memory retention (Girardeau et al. 2009; van de Ven et al. 2016; Norimoto et al. 2018). Preserved sequential firing during replay in conjunction with the electrophysiological properties of sharp‐wave/ripples suggests that neuronal plasticity emerging during sleep relies on Hebbian processes such as spike timing‐dependent plasticity (Sadowski et al. 2016; Maboudi et al. 2024). However, blocking AMPA or NMDA receptor‐mediated synaptic signalling, that is, the foremost neurochemical processes generating such plasticity, does not compromise the sleep‐dependent consolidation of declarative memory, that is, memory for facts and events (Feld et al. 2013). This raises the question of whether other neuromolecular mechanisms may support or even be essential for sleep‐induced plasticity. Combining experiments in healthy humans and rodents, we investigated whether these mechanisms involve the direct electrical coupling of neurons via gap junctions, that is, electrical synapses.

Gap junctions enable direct cell‐to‐cell exchange of electrical signals like ions via channels formed by two hemichannels, that is, connexons, which consist of six protein subunits or connexins (Salameh and Dhein 2005). The main connexin in the mammalian brain is connexin 36 (Cx36), which is predominantly expressed in neurons (Connors and Long 2004), particularly in the CA1, CA3 and CA4 subregions of the hippocampus (Condorelli et al. 1998). Cx36 channels are essential for the synchronization of rhythmic neuronal activity (Long et al. 2002). Thus, electrical coupling between neighbouring hippocampal neurons enables fast network oscillations (MacVicar and Edward Dudek 1981; Draguhn et al. 1998; Schmitz et al. 2001) and, notably, contributes to the generation of sharp‐wave/ripple complexes (Maier et al. 2003), which mark memory replay in the hippocampus (Diba and Buzsáki 2007). Cx36 deficiency reduces the occurrence of sharp‐wave and ripple oscillations in hippocampal slices (Maier et al. 2002), and Cx36 knockout mice not only display severe disruption of the synchronized activity of inhibitory neocortical networks (Deans et al. 2001), but also impaired motor‐coordination learning and impaired recognition memory (Frisch et al. 2005). Thus, the available evidence from animal models putatively links gap junctions to sleep's facilitating effect on memory consolidation (Rasch and Born 2013; Feld and Born 2020) and especially to the hallmarks of synchronized oscillatory activity of slow wave sleep (SWS), that is, hippocampal sharp‐wave/ripples as well as spindles and slow oscillations in the thalamo‐cortical system, which are well known to support memory formation during sleep (Staresina et al. 2015). Therefore, we tested the hypotheses that blocking electrical synapses (*i*) disrupts the consolidation of memory traces in the offline, sleeping brain, (*ii*) in particular affects the consolidation of declarative memory contents, whose formation recruits the hippocampus and adjacent structures (Zola‐Morgan and Squire 1990), and (*iii*) attenuates hippocampal sharp‐wave/ripple activity as a mechanism of memory replay.

Pharmacological options to block Cx36 gap junction channels in humans are restricted to the antimalarial agent quinine, the chemically closely related quinidine, and mefloquine, a synthetic quinolinemethanol, all of which have attracted scrutiny because of reports of side effects after prolonged intake. Quinine is used in cases of severe malaria and has been linked to vision and hearing loss (Bateman 2023), tinnitus (Tange et al. 1997) and hypoglycaemia (Limburg et al. 1993). While case reports have associated the use of mefloquine in malaria prevention and treatment with neuropsychiatric effects like vivid dreams, nightmares and vertigo (Juszczak and Swiergiel 2009), systematic reviews indicate a lower prevalence of side effects than seen with the other two substances (Bitta et al. 2017). The half maximal inhibitory concentration (IC_50_) of mefloquine for Cx36 is relatively low (0.3 μM; for Cx50, it is 1.1 μM) and can be achieved with a single oral dose of 250 mg (i.e., the weekly dose of preventive administration regimes; Cruikshank et al. 2004). Therefore, we expected this dose to reliably block Cx36‐dependent electrical coupling without substantial adverse reactions in the young, healthy male participants of our human study that tested hypotheses (*i*) and (*ii*). Before mefloquine administration, participants learned declarative word‐pair and 2‐D object‐location tasks and a procedural finger sequence task in the evening, which were retrieved after an interval of 20.5 h that included a night of sleep. Two control experiments were run to differentiate sleep‐specific effects on memory retention from effects that might also emerge during wakefulness or affect processes of memory retrieval. Because ethical considerations preclude intracranial recordings in healthy humans and administering mefloquine to patients undergoing intracranial electrophysiology, we also ran an experiment in rats to investigate the mechanistic hypothesis (*iii*). Here, we assessed the impact on hippocampal sharp‐wave/ripple activity of intraperitoneal (i.p.) mefloquine administration at the human‐equivalent dose of 20 mg/kg and an escalating dose of 40 mg/kg.

## 2 Materials and Methods

### 2.1 Experiments in Humans

#### 2.1.1 Participants

Our placebo‐controlled counter‐balanced within‐subject study in humans included a total of 44 participants (*experiment 1,* retention/sleep: *n* = 20, *experiment 2,* retention/wake: *n* = 12, *experiment 3,* retrieval: *n* = 12). They were healthy, nonsmoking, native German‐speaking men (18–30 years) who held degrees qualifying them for secondary education and were not taking prescribed medication. None of them reported any past or present chronic physical or psychological illness, and routine examinations prior to inclusion confirmed the absence of any acute mental or physical disease. Participants reported a normal sleep–wake pattern during the week preceding each session, did not work night‐shifts, and had not travelled across more than six time zones in the 12 weeks before participation. Participants were instructed to get up at 7:00 AM on the days of the experiments and not to ingest alcohol or (after 1:00 PM) caffeinated beverages. In experiments 1 and 3, an adaptation night was conducted at least 48 h before the actual experiment under identical conditions (i.e., including electrodes for polysomnographic recording). The experiments were approved by the local ethics committee (Ethics Committee of the Medical Faculty at the University of Tübingen). Written informed consent was obtained from all participants before participation and they were compensated financially.

#### 2.1.2 Design and Procedures

**FIGURE 1:**
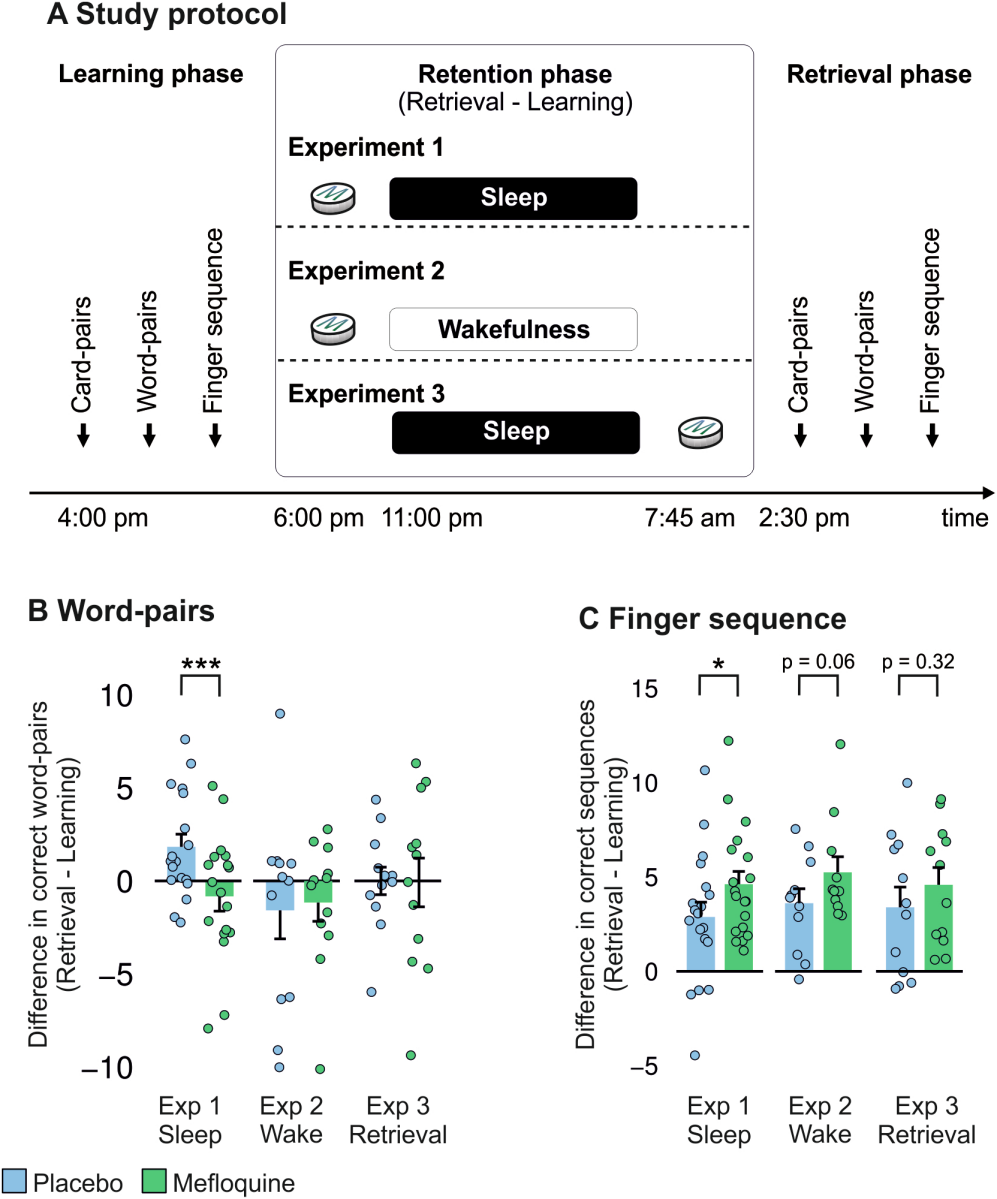
**Experiments in humans.** (A) Mefloquine (250 mg p.o.) and, respectively, placebo was administered to healthy participants after they had learned memory tasks (card pairs, word pairs, finger sequences) in the afternoon; recall was tested in the subsequent early afternoon. In experiment 1 (retention/sleep), participants received mefloquine or placebo at 6:00 PM. In experiment 2 (retention/wake), participants received the drug at the same time, but were not allowed to sleep until after retrieval. In experiment 3 (retrieval), participants were allowed to sleep, but received the drug after sleep. (B) Bar plot (mean and SEM) and individual data points (circles) of retention performance for word pairs in the mefloquine condition (green) and the placebo condition (blue) of the individual experiments. Retention was calculated as (*number of word pairs correctly recalled at retrieval ‐ number of word pairs correctly recalled at learning*). (C) Bar plot (mean and SEM) and individual data points (circles) of retention performance in the finger‐sequence task for the mefloquine condition (green) and the placebo condition (blue) of the individual experiments, which was calculated as (*average retrieval performance ‐ average learning performance*).

All three experiments followed a double‐blind, placebo‐controlled, within‐subject, balanced crossover design (see Figure 1A in paragraph 3.2 for an overview). Each experiment consisted of two experimental sessions that were conducted at least 4 weeks apart and used an identical set‐up including parallel versions of the learning tasks. In counter‐balanced order, participants received 250‐mg mefloquine (Lariam, plasma peak: 10 h, half‐life: 10 days; Gbotosho et al. 2012) in one and placebo in the other session. Identical capsules containing mefloquine or placebo and manufactured according to GMP were prepared by the pharmacy at the University Medical Centre of the University of Mainz, Germany.

In general, participants arrived at the lab at 4:00 PM to start the learning phase (see paragraph 2.1.3 for details). They always learned the tasks in the sequence of card pairs, word pairs and finger sequence tapping with 10‐min breaks of playing a computer game (Snood) in‐between. After learning, vigilance, mood and sleepiness were assessed (see 2.1.4) and the participant received a snack (a traditional Swabian pretzel with butter) to enhance tolerability of mefloquine intake and watched animal documentaries (BBC’s ‘Planet Earth’). In experiments 1 and 2, the drug was administered at 6:00 PM, thus ensuring sufficient blood concentrations at the beginning of the sleep or sleep‐deprivation period (Gbotosho et al. 2012) without scheduling the learning phase too far from sleep onset time in the sleep group (Gais et al. 2006). Participants afterwards continued watching documentaries and, at 7:00 PM, received a standardized dinner (two slices of bread, butter, cheese and/or ham, tomato and decaffeinated tea).

Electrodes for polysomnography were applied at 9:00 PM in experiments 1 and 3. At 10:15 pm blood was sampled for the assessment of mefloquine and the participant filled in questionnaires on mood and sleepiness. In these experiments, the electrodes were connected to the amplifier and lights were turned off at 11:00 PM. In experiment 2, participants remained awake throughout the night and watched animal documentaries (see above). Participants of experiments 1 and 3 were woken up between 6:45 and 7:15 AM. Participants of all experiments filled in questionnaires on mood and sleepiness and blood was sampled at 7:30 AM. Participants of experiment 3 received the drug at 7:45 AM (to induce high concentrations at retrieval of the memory contents). All participants left the lab in the morning and spent the time until retrieval as they pleased. They were however instructed not to sleep, to study or to consume caffeine or alcohol. Participants of experiment 2 were continuously accompanied by an investigator to ensure wakefulness. They all returned to the lab at 2:30 pm and the memory tasks were retrieved in the same order as during learning (card pairs, word pairs and finger sequence). Afterwards, participants performed memory control tasks (novel finger sequence tapping and number encoding, see 2.1.3.4 and 2.1.3.3). Subsequently, general retrieval function, vigilance, mood and sleepiness were assessed, and the participant was asked if he assumed to have received mefloquine or placebo. Finally, a last blood sample was collected. We did not find indicators in any of the experiments that participants were able to identify the condition they were in (mefloquine or placebo; all *p* ≤ 0.34, exact McNemar's test).

#### 2.1.3 Memory Tasks

##### 2.1.3.1 Card Pairs

The declarative card‐pair task is similar to the game ‘concentration’ inasmuch as the participants learn 15 card pairs arranged in a 5 × 6 matrix on a computer screen and depicting animals and everyday objects (Rasch et al. 2007; Diekelmann et al. 2011). During the learning phase, the first card of each pair was shown alone for 1 s, followed by the 3‐s presentation of the complete card pair with an interstimulus interval of 3 s. The whole set of card pairs was shown twice in different orders. A cued recall procedure was conducted next, in which the participant was shown the first card of a pair and had to click on the corresponding second card with a computer mouse. Independent of the response, the correct card was shown for 2 s afterward. This cued recall procedure was repeated until the participant reached the criterion of 60% percent correct answers. During retrieval the same cued retrieval procedure was used. Retention performance was calculated as (*number of card‐pairs correctly recalled at retrieval ‐ number of card‐pairs correctly recalled at learning*), reflecting the ability to maintain encoded information over time and, thereby, memory retention.

##### 2.1.3.2 Word Pairs and Word Generation

In the word‐pair task to assess declarative memory performance (Ekstrand et al. 1977; Plihal and Born 1997), participants started learning by seeing each of the 40 word pairs for 4 s with an interstimulus interval of one second. Immediately afterwards, recall was tested in a cued recall procedure by showing the first word of each pair and asking the participant to say the matching second word. Irrespective of his answer, the participant afterwards received feedback on the correct word for 2 s. This cued recall procedure was repeated until the participant reached the criterion of 60% correct responses. During retrieval, the cued recall procedure was repeated without feedback. Retention performance was calculated as (*number of word pairs correctly recalled at retrieval ‐ number of word pairs correctly recalled at learning*). In the word generation task performed during retrieval (Regensburg verbal fluency test; Aschenbrenner et al. 2000), participants were given 2 min each to write down as many words as possible that started with the cue letter *p* or *m*, or belonged to the cue category *hobby* or *profession*. Sum scores of both versions of the task were regarded as a measure of general retrieval performance.

##### 2.1.3.3 Number Encoding

During retrieval participants also learned 16 three‐digit numbers presented on a computer screen for 2 s (500‐ms interstimulus interval) in three random blocks (Feld et al. 2013). After a short break of about 1 min, participants were asked to freely recall the numbers and write them on a piece of paper, and we analyzed the amount of correct numbers. Afterwards, they were again shown the previously learned numbers, but intermixed with 16 new numbers, and had to decide which numbers they had already learned. From the hit rate and the false alarm rate, we calculated the sensitivity index d‐prime to measure recognition memory.

##### 2.1.3.4 Finger Sequence Tapping

The finger sequence tapping task measures procedural memory performance (Walker et al. 2003). During learning, participants tapped a five‐digit finger sequence (e.g., 4–1–3–2–4) as fast and as accurately as possible during twelve 30‐s blocks that were interrupted by 30‐s breaks. To keep working memory demands at a minimum, the sequence was displayed on the screen at all times and an asterisk displayed under the current number was used to signal a button press. Responses were scored for speed (number of correctly completed sequences) and errors, and this information was presented to the participant after every 30‐s block. The final three blocks of the learning phase were averaged to indicate learning performance. During the retrieval phase, participants performed on an additional three blocks of the task, and these were averaged to indicate retrieval performance. Performance speed was assessed as the total amount of correctly tapped sequences and performance accuracy as the error rate per block. Retention performance was calculated as (*average retrieval performance ‐ average performance in the final three blocks of learning*). After retrieval, participants performed on an additional three blocks of a new sequence to assess if potential performance changes at retrieval were due to memory‐independent effects like unspecific changes in reaction speed.

#### 2.1.4 Control Measures: Sleepiness, Vigilance and Mood

Subjective sleepiness was measured using the one‐item Stanford Sleepiness Scale (SSS; Hoddes et al. 1973) during the learning and retrieval phases, as well as in the evening and the morning. Here, participants indicated how sleepy they felt on a scale ranging from one (‘Feeling active, vital, alert or wide awake’) to eight (‘Asleep’—provided as an anchor). Objective vigilance was assessed by means of the psychomotor vigilance task (PVT; Dinges et al. 1997) at the end of the learning and retrieval phases. In this task, the participants saw a red millisecond counter at the centre of a black screen that occasionally and suddenly started counting upwards from 0. Pressing the response button stopped the counter and yielded the reaction time. Participants were asked to react as fast as possible during 5 min and average reaction speed (one divided by reaction time) was used as the objective measure of vigilance. We assessed the mood of our participants by means of a multidimensional mood questionnaire at four time points per session (Steyer et al. 1997). This questionnaire comprises the three dimensions mood (high values reflect positive mood), tiredness (low values equal tiredness) and calmness (high values reflect calmness).

#### 2.1.5 Sleep Scoring and Frequency Analyses

Standard polysomnography in experiments 1 and 3 was performed using Ag‐AgCl cup electrodes connected to a Brain Amp DC 32‐channel EEG‐amplifier (Brain Products GmbH, Gilching, Germany) for the EEG channels and a Brain Amp ExG 8‐channel bipolar amplifier (Brain Products GmbH, Gilching, Germany) for the bipolar EOG and EMG channels that shared a ground electrode connected to the forehead. EEG was recorded from F3, Fz, F4, C3, Cz, C4, P3, Pz, P4 according to the international 10–20 system and referenced to coupled electrodes attached to the mastoids. Additionally, horizontal and vertical eye movements and muscle tone (from the chin) were recorded. Heart rate was recorded via ECG electrodes to monitor for adverse side effects. Data were low‐pass‐ (80 Hz) and high‐pass‐filtered (0.16 Hz) before digitizing at 250 Hz. For sleep scoring, they were down‐sampled to 200 Hz and additional offline low‐pass (35 Hz) and high‐pass (0.32 Hz) filters were applied. Sleep stages were scored according to Rechtschaffen and Kales (1968) using C3 and C4 by two experienced investigators who were blind to the assigned condition. Differences in scoring between the scorers were resolved by consulting a third experienced investigator.

Off‐line detection of slow oscillations was based on an algorithm described in detail previously (Mölle et al. 2002) and was applied to SWS epochs across the entire night. In brief, the EEG for each channel was first low‐pass‐filtered at 30 Hz and down‐sampled to 100 Hz. For the identification of large slow oscillations, a low‐pass filter of 3.5 Hz was applied. Then, negative and positive peak potentials were derived from all intervals between consecutive positive‐to‐negative zero crossings (i.e., one negative and one positive peak for every interval). Only intervals with durations of 0.833–2 s (corresponding to a frequency of 0.5–1.2 Hz) were included. We determined each participant’s individual detection thresholds by first calculating the mean values of the negative peak potential and the negative‐to‐positive peak amplitude of the valid intervals within each channel. These were then averaged across all nine EEG channels. Subsequently, we marked for each channel those intervals as slow oscillation epochs in which the negative peak amplitude was lower than the mean negative peak threshold multiplied by 1.25, and where the amplitude difference (positive peak minus negative peak) was larger than the peak‐to‐peak amplitude threshold multiplied by 1.25. Negative half‐wave peaks were used to characterize slow‐oscillation events. Time‐frequency plots were derived using the Fieldtrip toolbox for Matlab (http://www.fieldtriptoolbox.org, ‘mtmconvol’ function) on ± 1.5‐s windows time‐locked to the negative peak of offline‐detected slow‐oscillation events for frequencies from 5 to 20 Hz in steps of 0.5 Hz, using a Hanning window corresponding to seven cycles for each frequency, which was further shifted along the time axis in steps of 40 ms. The resulting time‐frequency representations were normalized to a pre‐event baseline from −1.5 to −1.4 s.

For statistical comparisons between conditions, we selected samples that showed significant differences in relative power based on two‐tailed paired *t* tests (sample‐level alpha = 0.05). In the resulting statistical map, adjacent samples were grouped into positive and negative clusters, for which cluster‐level statistics were calculated by summing up the t‐values within each cluster. These were tested against a reference distribution (cluster‐level alpha = 0.05), generated by shuffling the association of data and condition (1000 permutations) and, for each permutation, taking the maximum statistics among all clusters.

#### 2.1.6 Serum Mefloquine Concentrations

Blood was sampled three times (in the evening, in the morning and after the retrieval phase) for the determination of mefloquine concentrations. Blood was centrifuged after 10 min of incubation at room temperature, and serum samples were frozen at −80°C. Mefloquine levels were measured by high‐performance liquid chromatographic analysis with a detection limit of 20 ng/mL, an interassay coefficient of variation (CV) of 3.28% and an intra‐assay CV of 6.52% (Dr. Eberhard & Partner, Dortmund, Germany). Any data points below the detection limit were considered to be 0. Blood samples collected in one placebo session of experiment 3 were lost, but respective values set to 0 because it was this participant's first experimental session. The third blood sample of the mefloquine session of one individual of experiment 2 was likewise lost, so that this participant's data were omitted from the analysis of this time point.

#### 2.1.7 Statistical Analyses

One participant of experiment 1 was excluded from data analyses because of insufficient sleep in the placebo night. Word‐pair data of another participant of experiment 1 were discarded because of retention performance of more than 2 standard deviations below the group mean. Finger sequence tapping data of one participant of experiment 2 were excluded because of extreme underperformance during the last three blocks of the learning phase (compared to the rest of the learning) in one condition reflecting underrepresentation of encoding. Psychomotor vigilance task data of two participants (from experiments 2 and 3) were discarded because they indicated a misconception of the instructions (repeated responses in the absence of cues); one time point of assessment was lost in experiment 1 due to technical malfunction.

Statistical analyses generally relied on analyses of covariance with the factors condition (mefloquine vs. placebo), time point and experiment (1, 2, 3) as appropriate. Since mefloquine was detectable in samples collected in the placebo session of six, three and three participants in experiments 1, 2 and 3, respectively, which constitutes roughly 50% of cases in which the placebo session took place after the mefloquine session (see Supplementary Table S1 for descriptive data), serum mefloquine values during the learning phase of the placebo session were included as covariates. (In the analyses of the number encoding task, mefloquine levels during the retrieval rather than learning phase were used as covariates.) Note that the mefloquine‐induced impairment of declarative memory consolidation reported in 3.2 is also found in analyses conducted without the covariate. Greenhouse–Geisser correction of degrees of freedom was performed whenever necessary. Control measures were compared by paired *t* tests at each time point to detect intra‐experimental differences unless specified otherwise. Two‐sided tests were used unless explicitly specified otherwise.

### 2.2 Experiments in Animals

#### 2.2.1 Animals, Study Design and Procedure

We performed experiments in rats to test our hypothesis that mefloquine attenuates hippocampal sharp‐wave/ripple activity, recording local field potentials (LFPs) in the hippocampus as well as frontal and parietal cortical EEG before and after i.p. administration of placebo and mefloquine. Mefloquine was administered at the human‐equivalent dose of 20 mg/kg (Nair and Jacob 2016), which corresponds to comparable experiments in rats (Bissiere et al. 2011), and an escalating dose of 40 mg/kg (see Figure 3A in paragraph 3.6 for an overview of the experimental design). Male Long Evans rats (Janvier, Le Genes‐Saint‐Isle, France; *n* = 6, 320–334 g, 16–17 weeks old) were used. The rats were kept in temperature‐ (22°C ± 2°C) and humidity‐ (45%–65%) controlled cages, on a 12‐h light/dark cycle with the lights off at 19:00 h. Water and food were available ad libitum. All experimental procedures were approved by the Baden‐Württemberg state authority in charge of animal welfare (Regierungspräsidium Tübingen).

After the animals had been handled for 7 days, electrodes for the recording of EEG, EMG and LFPs were surgically implanted (see below) and animals recovered for another 7 days before being habituated to the recording box (dark grey PVC, 30 × 30 cm, height: 40 cm) for 3 days (3 h per day). Subsequently, 12‐h baseline recordings were performed from 7:00 AM to 7:00 PM, and at 7:00 AM of the subsequent day, the animals received placebo (1 mL i.p. of 20% dimethyl sulfoxide [DMSO] in 0.9% saline). Two postadministration 12‐h recordings followed immediately afterwards and, respectively, on the subsequent day (again from 7:00 AM to 7:00 PM). After a pause of 3 days, the procedure was repeated twice, but instead of placebo, the animals were injected mefloquine at doses of, first, 20 mg/kg and, after another pause of 3 days, 40 mg/kg body weight. For the injections, mefloquine hydrochloride (Sigma‐Aldrich, St. Louis, USA) was dissolved in DMSO for a 38 mg/mL stock solution and diluted in 0.9% saline. During the recordings, the animal's behaviour was continuously tracked using a video camera mounted on the recording box, and the electrodes were connected by a swivelling commutator to an amplifier (Digital Lynx SX, Neuralynx Inc., Bozeman, USA). EEG, LFP and EMG signals were continuously recorded using the Cheetah software (Neuralynx Inc., Bozeman, USA). EEG signals were amplified and filtered between 0.1 and 300 Hz. LFP signals were amplified and filtered between 0.1–1000 Hz. EMG signals were filtered between 30 and 300 Hz. All signals were sampled at 1 kHz. At the end of the experiment, animals were perfused under deep anaesthesia.

#### 2.2.2 Surgery

Animals were anaesthetised with an i.p. injection of fentanyl (0.005 mg/kg body weight), midazolam (2 mg/kg) and medetomidin (0.15 mg/kg). They were placed into a stereotaxic frame and were supplemented with isoflurane (0.8%–1.2%) if necessary. The scalp was exposed and five holes were drilled into the skull. Three EEG screw electrodes were implanted: one frontal electrode (AP: +2.6 mm, ML: −1.5 mm, relative to Bregma), one parietal electrode (AP: −2.0 mm, ML: −2.5 mm, relative to Bregma) and one occipital reference electrode (AP: −10.0 mm, ML: 0.0 mm, relative to Bregma). Additionally, a silicon probe (NeuroNexus, Ann Arbor, USA) was implanted to record LFP signals in the right hippocampus (AP: −3.1 mm, ML: +3.0, DV: −3.6 mm). Electrode positions were confirmed by histological analysis. For EMG recordings, a stainless‐steel wire was implanted in the neck muscle. Electrodes were connected to a six‐channel connector (Mill‐Max, USA) and fixed with cold polymerizing dental resin, and the wound was sutured.

#### 2.2.3 Analyses of EEG, LFP and EMG Recordings

##### 2.2.3.1 Sleep Stage Determination

Sleep stages (SWS and rapid eye movement sleep, REM sleep) and wakefulness were determined off‐line based on EEG and EMG recordings, using standard visual scoring procedures for consecutive 10‐s epochs as previously described (Neckelmann et al. 1994; Durán et al. 2018). Wakefulness was identified by mixed‐frequency EEG and sustained EMG activity, SWS by the presence of high amplitude low activity (delta activity < 4.0 Hz) and reduced EMG tone, REM sleep by low‐amplitude EEG activity with predominant theta activity (5.0–10.0 Hz), phasic muscle twitches and decrease of EMG tone.

##### 2.2.3.2 Ripple Detection

Ripples were identified in the left dorsal hippocampal LFP recordings as described previously (Mölle et al. 2006). The signal was filtered between 80.0 and 250.0 Hz. A ripple event was identified when the Hilbert transform of the filtered signal exceeded a threshold of 2.0 SDs from the mean signal across all SWS epochs of the respective recording session for at least 30 ms (including at least 3 cycles) and for not more than 300 ms, and the maximum amplitude of the filtered signal reached at least 5.0 SDs. The median frequency of ripples was determined using wavelet analysis in the FieldTrip toolbox (Oostenveld et al. 2011) as follows: Initially, frequency analysis was conducted for each detected ripple event within the 80.0‐ to 250.0‐Hz range, with 1.0‐Hz increments. This analysis employed a sliding Hanning taper time window ranging from −0.5 to +0.5 s, with steps of 0.05 s relative to each ripple's onset. Subsequently, the data at each time point was normalized by dividing the values during the ripple event by the mean amplitude of each frequency band during a baseline time window from −0.3 to 0 s relative to the ripples. The normalization procedure utilized the FieldTrip function ‘ft_freqbaseline’ with ‘baselinetype’ set to ‘relative’. After normalization, the frequency of each ripple event was determined by calculating the median of the normalized frequency distribution for each ripple event. Finally, the peak in the resulting distribution was identified as the median frequency of the respective recording session.

#### 2.2.4 Statistical Analyses

Data obtained after the injection of, respectively, placebo and mefloquine were averaged across the two postadministration 12‐h intervals for comparisons with the 12‐h preadministration baselines. Baseline and postadministration data were submitted to analyses of variance (ANOVA) with the repeated measures factors drug (mefloquine vs. placebo) and time (baseline vs. postadministration), followed by post hoc paired sample *t* tests.

## 3 Results

### 3.1 Summary of Findings

In the experiments in humans, mefloquine administered to block Cx36 gap junction channels impaired the consolidation of declarative memory contents in a sleep‐specific fashion and improved procedural memory retention irrespective of sleep. On the neurophysiological level, mefloquine disrupted the coupling of sleep spindles to slow oscillations, hallmarks of NREM sleep. Contrary to expectations, mefloquine did not affect hippocampal sharp‐wave/ripple activity in rats, suggesting that the contribution of electrical coupling to memory formation rather involves extrahippocampal mechanisms of rhythmic neuronal activity.

### 3.2 Mefloquine Disrupts the Sleep‐Dependent Retention of Word‐Pair Memory in Humans

The word‐pair task robustly reflects sleep‐dependent consolidation of declarative memory contents (Ekstrand et al. 1977; Plihal and Born 1997). Mefloquine compared to placebo impaired retention of word pairs across the consolidation interval in the sleeping subjects of experiment 1, *retention/sleep* (mefloquine: −0.83 (0.79), placebo: 1.83 (0.68) word pairs, *F*_(1,16)_ = 29.75, *p* ≤ 0.001, η_p_^2^ = 0.65; see Table 1 for descriptive data). This effect was not observed in the *retention/wake* experiment 2 (mefloquine: −1.17 (1.00), placebo: −1.58 (1.53), *F*_(1,10)_ = 2.43, *p* = 0.15, η_p_^2^ = 0.20) and the *retrieval* experiment 3 (mefloquine: −0.08 (1.31), placebo: 0.00 (0.74), *F*_(1,10)_ = 0.14, *p* = 0.72, η_p_^2^ = 0.014; Figure 1B). This pattern was statistically corroborated by an overall experiment × condition interaction effect (*F*_(1,39)_ = 4.52, *p* = 0.040, η_p_^2^ = 0.10), indicating that mefloquine reduced retention performance for word pairs exclusively in the sleep experiment. Learning performance (reflected by the number of word pairs correctly recalled at the end of encoding) appeared to be somewhat weaker in the placebo condition of experiment 1 than in the placebo conditions of experiments 2 and 3—albeit on a nonsignificant level (all *p* ≥ 0.11, independent *t* tests). Therefore, we conducted additional analyses of memory retention across experiments, in which we included only the 12 participants of experiment 1 whose learning performance was most comparable to that of the participants of experiment 3. This analysis confirmed our primary analysis, that is, yielded reduced memory retention upon mefloquine versus placebo administration in experiment 1 (*p* = 0.03) and a significant experiment × condition interaction (*F*_(1,33)_ = 4.10, *p* = 0.05, η_p_^2^ = 0.11). The beneficial effect on declarative memory retention of sleep per se was corroborated in a comparison covering the placebo conditions of the three experiments (*F*_(1,39)_ = 4.41, *p* = 0.042, η_p_^2^ = 0.10), demonstrating that the effect, whose magnitude was well in line with comparable studies in humans (Feld et al. 2013, 2014; Lustenberger et al. 2015), was not affected by the relatively long learning‐to‐sleep delay used here.

We also tested verbal fluency to check whether mefloquine affects general retrieval performance of remote memories that can be expected to be completely consolidated. Results did not indicate differences between mefloquine and placebo in any of the experiments (all *p* ≥ 0.10; see Supplementary Table S1 for descriptive data). Comparing the individual experiments, however, we found a main impairing effect of sleep deprivation (*F*_(1,41)_ = 4.78, *p* = 0.035, η_p_^2^ = 0.10). Analyses of the placebo conditions in terms of learning performance during the retrieval phase (three‐digit number task) showed that sleep compared to wakefulness in general enhanced recognition (*F*_(1,40)_ = 6.06, *p* = 0.018, η_p_^2^ = 0.13) but not free recall (*F*_(1,40)_ = 1.88, *p* = 0.178, η_p_^2^ = 0.05; see Table 1 for descriptive data), which accords with previous reports of improved encoding after sleep (Mander et al. 2011). We did not observe detrimental effects of mefloquine on encoding. If at all, there was an improvement in recognition under mefloquine in the wake experiment (*F*_(1,10)_ = 7.20, *p* = 0.023, η_p_^2^ = 0.42) that was not evident in the sleep (*F*_(1,17)_ = 0.01, *p* = 0.91, η_p_^2^ ≤ 0.01) or retrieval experiment (*F*_(__1,10)_ = 1.79, *p* = 0.210, η_p_^2^ = 0.15). This pattern was reflected in a trend‐wise statistical interaction (main effect condition: *F*_(1,39)_ = 4.56, *p* = 0.039, η_p_^2^ = 0.11, condition × experiment: *F*_(2,39)_ = 3.02, *p* = 0.060, η_p_^2^ = 0.13) and might reflect vigilance‐promoting effects of mefloquine, which can be expected to be most prominent after sleep deprivation, as likewise suggested by control measures (see 3.5).

**TABLE 1:** Mean (standard error of the mean, SEM) performance on the memory tasks, that is, correctly recalled word pairs, correctly tapped sequences, accuracy of tapped sequences, correctly recalled card‐pair locations and numbers recalled and recognized. Retention performance is retrieval performance minus learning performance; percent of learning is absolute retrieval score divided by learning score.

	Experiment 1 (retention/sleep)				Experiment 2 (retention/wake)				Experiment 3 (retrieval)			
	Mefloquine		Placebo		Mefloquine		Placebo		Mefloquine		Placebo	
**Word pairs**												
Learning	29.11	(0.93)	27.83	(0.80)	28.75	(1.08)	29.17	(1.01)	29.83	(0.89)	30.00	(1.10)
Retrieval	28.28	(1.17)	29.67	(1.09)	27.58	(1.67)	27.58	(1.65)	29.75	(1.21)	30.00	(1.18)
Retention performance	−0.83	(0.79)	1.83	(0.68)	−1.17	(1.01)	−1.58	(1.53)	−0.08	(1.31)	0.00	(0.74)
Percent of learning	0.97	(0.03)	1.07	(0.02)	0.95	(0.04)	0.95	(0.05)	1.00	(0.05)	1.00	(0.03)
Trials to criterion	1.50	(0.19)	1.67	(0.16)	1.75	(0.18)	1.67	(0.14)	1.33	(0.19)	1.33	(0.14)
**Finger sequence**												
*Correct sequences*												
Learning	20.79	(0.91)	22.18	(1.02)	18.64	(1.33)	20.67	(1.32)	20.58	(1.55)	21.75	(1.58)
Retrieval	25.40	(1.25)	25.05	(1.28)	23.88	(1.72)	24.27	(1.60)	25.17	(2.21)	25.14	(2.07)
Retention performance	4.61	(0.67)	2.88	(0.78)	5.24	(0.83)	3.61	(0.77)	4.58	(0.91)	3.39	(1.07)
Percent of learning	1.22	(0.03)	1.13	(0.03)	1.29	(0.04)	1.18	(0.04)	1.22	(0.04)	1.15	(0.05)
Control sequence	16.63	(1.03)	16.05	(0.89)	16.30	(1.13)	15.70	(1.21)	16.89	(1.81)	18.44	(1.86)
*Error rates*												
Learning	0.10	(0.01)	0.07	(0.01)	0.13	(0.03)	0.10	(0.01)	0.14	(0.02)	0.09	(0.01)
Retrieval	0.07	(0.02)	0.08	(0.01)	0.03	(0.01)	0.06	(0.01)	0.09	(0.02)	0.10	(0.02)
Retention performance	−0.03	(0.01)	0.00	(0.02)	−0.10	(0.02)	−0.04	(0.01)	−0.05	(0.02)	0.01	(0.02)
Control sequence	0.14	(0.02)	0.14	(0.03)	0.05	(0.01)	0.12	(0.02)	0.14	(0.04)	0.11	(0.02)
**Card pairs**												
Learning	0.72	(0.02)	0.68	(0.02)	0.72	(0.03)	0.68	(0.02)	0.70	(0.02)	0.70	(0.03)
Retrieval	0.55	(0.03)	0.53	(0.03)	0.61	(0.06)	0.56	(0.02)	0.55	(0.04)	0.54	(0.06)
Retention performance	−0.16	(0.03)	−0.15	(0.03)	−0.11	(0.06)	−0.12	(0.02)	−0.15	(0.04)	−0.16	(0.05)
Trials to criterion	3.00	(0.47)	3.16	(0.45)	2.25	(0.30)	2.42	(0.43)	2.00	(0.39)	2.67	(0.50)
**Numbers**												
Free recall												
Correct	7.37	(0.86)	8.16	(0.69)	7.92	(1.03)	6.42	(0.78)	7.67	(0.57)	6.92	(0.53)
False	3.95	(0.86)	4.05	(0.57)	2.83	(0.61)	3.75	(1.08)	3.00	(0.49)	3.17	(0.49)
Percent correct	0.65	(0.06)	0.67	(0.04)	0.72	(0.06)	0.66	(0.07)	0.72	(0.04)	0.69	(0.04)
Recognition												
Hits	0.78	(0.04)	0.85	(0.03)	0.76	(0.05)	0.67	(0.05)	0.72	(0.04)	0.69	(0.04)
False alarms	0.28	(0.03)	0.24	(0.03)	0.26	(0.02)	0.30	(0.05)	0.16	(0.03)	0.21	(0.04)
d‐prime	1.49	(0.22)	1.82	(0.19)	1.47	(0.22)	1.07	(0.19)	1.70	(0.20)	1.40	(0.19)

We also tested our participants on a two‐dimensional object location task (card pairs), which has been previously used to investigate reactivation of memory by external cues during sleep (Rasch et al. 2007; Diekelmann et al. 2011), but in the absence of external cuing shows less distinct enhancements across sleep than word‐pair memories (Ekstrand et al. 1977; Plihal and Born 1997; Diekelmann et al. 2012). Therefore, the task was also included in an exploratory fashion to test its suitability for future studies on the role of gap junctions in exogenously cued memory replay. We found no differences between conditions in encoding performance (all *p* ≥ 0.11, see Table 1 for descriptive data), nor an improving effect of sleep on retention performance in the placebo conditions (*F*_(1,40)_ = 0.57, *p* = 0.45, η_p_^2^ = 0.01), which is in line with comparable studies (Rasch et al. 2007; Diekelmann et al. 2012). There was no evidence of mefloquine effects on retention performance in analyses covering the individual (all *p* ≥ 0.56) or all three experiments (main effect condition: *F*_(1,39)_ = 1.40, *p* = 0.24, η_p_^2^ = 0.04, condition × experiment: *F*_(2,39)_ = 0.34, *p* = 0.72, η_p_^2^ = 0.02). This outcome may have been due to the long learning‐to‐sleep delay, which was necessary to enable optimal uptake of the drug, leading to floor effects and thereby further masking memory‐enhancing effects of sleep. Hence, other cueing tasks that are more robust regarding learning‐to‐sleep delays may be considered for subsequent memory studies on the impact of mefloquine (Schreiner and Rasch 2015; Schreiner et al. 2015).

### 3.3 Mefloquine Improves the Consolidation of Procedural Memory Irrespective of Sleep

Encoding performance on the procedural memory task, that is, the amount of correctly tapped sequences in the last three blocks of the learning phase, was comparable across experiments and conditions (all *p* ≥ 0.14; Table 1). Overall analyses revealed a mefloquine‐induced improvement of finger sequence retention, that is, more correctly tapped sequences, that emerged irrespective of the experiment (main effect condition, *F*_(1,38)_ = 3.97, *p* = 0.054, η_p_^2^ = 0.10; condition × experiment, *F*_(2,38)_ = 0.09, *p* = 0.91, η_p_^2^ = 0.01; Figure 1C). Performance accuracy as reflected by error rates, that is, incorrectly tapped sequences divided by total tapped sequences, showed an even more pronounced general improvement across the retention interval under mefloquine (main effect condition, *F*_(1,38)_ = 8.74, *p* = 0.005, η_p_^2^ = 0.19; condition × experiment, *F*_(2,38)_ = 0.65, *p* = 0.53, η_p_^2^ = 0.03). Comparing retention performance between the placebo conditions, we did not observe a beneficial effect of sleep on tapping performance (*F*_(1,39)_ = 0.22, *p* = 0.65, η_p_^2^ = 0.01). The absence of a clear consolidation benefit of sleep per se for procedural memory may, again, have been due to the extended period of wake retention before sleep (Nettersheim et al. 2015), but also adds to the ongoing debate surrounding the role of sleep for motor memory consolidation (Pan and Rickard 2015; Adi‐Japha and Karni 2016; Rickard and Pan 2017; Rickard et al. 2022). Performance on a control finger sequence, tested exclusively at retrieval, remained unaffected by the drug (all *p* ≥ 0.12 for number of correct sequences and error rate).

### 3.4 Mefloquine Does Not Alter Sleep Architecture but Affects NREM Sleep Physiology

**FIGURE 2:**
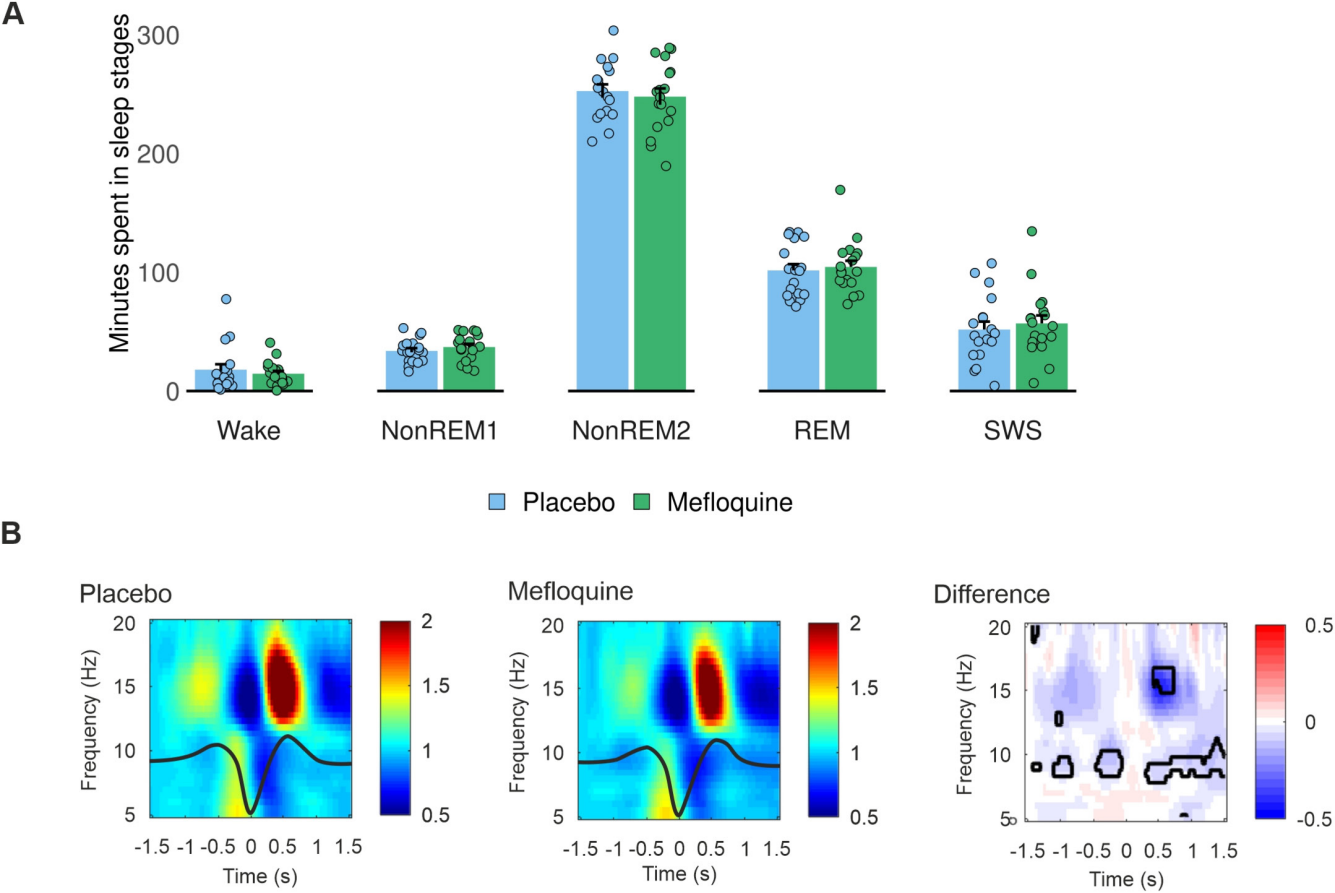
**Sleep results of experiment 1.** (A) Bar plot (mean and SEM), and individual data points (circles) of time spent in different sleep stages (SWS = slow wave sleep, REM = rapid eye movement sleep) for the mefloquine condition (green) and the placebo condition (blue) (see Table 2 for sleep results of experiment 3). (B) Effects of mefloquine on the coupling of sleep spindles to slow oscillations (0.75 Hz) were analysed by identifying the peaks of the slow oscillation down‐states and calculating EEG power between 5 and 20 Hz in 3‐s time windows centered on the down‐states (x‐axis: ‘0’). The left and middle panels depict this time‐frequency representation for the placebo and the mefloquine conditions with the grand average of the detected slow oscillations overlaid as thick solid lines. The right panel depicts the difference between the two conditions, with the areas of statistically significant differences outlined in black (two‐tailed paired‐samples *t* tests corrected for multiple comparisons). Colour bars to the right of the panels indicate scales of spectral power relative to the average power obtained between −1.5 and −1.4 s with respect to the slow oscillation down‐state (left and middle panel) and of respective differences between conditions (right panel). (Recordings obtained from EEG channel Pz are shown; see Supplementary Figure S1 for Fz and Cz.)

Mefloquine administration before sleep in experiment 1 had no effect on gross sleep architecture (all *p* ≥ 0.23, see Figure 2A and Table 2 for descriptive data). As expected, no differences between the sleep conditions were observed when mefloquine was administered after sleep in experiment 3 (all *p* ≥ 0.16). In more fine‐grained analyses of the results of experiment 1, we explored potential mefloquine effects on the relationship between slow oscillations (0.75 Hz) and sleep spindles, that is, the most prominent features of NREM sleep to benefit memory function (Clemens et al. 2007; Staresina et al. 2015). In parietal regions, mefloquine reduced power in the fast spindle band (12–15 Hz) during the up‐state of the slow oscillation and, moreover, decreased power in the slow spindle band (9–12 Hz) during the respective up‐ to down‐state transition (Figure 2B). These effects were less pronounced or absent at frontal and central electrode leads (Supplementary Figure S1).

**TABLE 2:** Mean (SEM) of the sleep stages scored according to standard criteria.

	Experiment 1 (retention/sleep)				Experiment 3 (retrieval)			
	Mefloquine		Placebo		Mefloquine		Placebo	
**Percent**								
Wake	3.15	(0.51)	3.88	(0.96)	3.91	(1.04)	1.98	(0.50)
NREM 1	8.03	(0.59)	7.37	(0.53)	6.51	(0.89)	5.57	(0.60)
NREM 2	53.47	(1.49)	54.90	(1.31)	55.17	(1.40)	55.30	(0.73)
NREM 3	9.64	(0.78)	8.93	(0.94)	8.69	(0.82)	9.99	(0.64)
NREM 4	2.53	(1.24)	2.19	(1.04)	2.64	(0.67)	2.29	(0.72)
SWS	12.18	(1.40)	11.12	(1.41)	11.33	(1.22)	12.29	(0.88)
REM	22.56	(1.12)	22.03	(1.09)	21.90	(1.11)	23.56	(1.32)
**Minutes**								
TST	463.81	(4.42)	461.00	(5.76)	471.63	(2.79)	471.83	(2.68)
Wake	14.58	(2.36)	17.97	(4.56)	18.50	(4.97)	9.25	(2.37)
NREM 1	37.06	(2.65)	33.78	(2.35)	30.71	(4.18)	26.33	(2.88)
NREM 2	247.92	(6.91)	252.61	(5.63)	260.17	(6.83)	261.00	(3.96)
NREM 3	44.94	(3.71)	41.44	(4.44)	41.00	(3.83)	47.17	(3.13)
NREM 4	11.97	(5.90)	10.33	(4.93)	12.50	(3.14)	11.00	(3.46)
SWS	56.92	(6.75)	51.78	(6.68)	53.50	(5.71)	58.17	(4.36)
REM	104.50	(5.20)	101.58	(5.30)	103.08	(5.01)	110.92	(5.80)
Movement time	2.83	(0.35)	3.28	(0.34)	5.67	(0.72)	6.17	(0.52)

### 3.5 Mefloquine Enhances Vigilance After Sleep Deprivation

During the learning and the retrieval phase as well as in the evening and the morning, we used questionnaire and performance measures to detect potential effects of mefloquine on alertness and mood (see Supplementary Table S1 for descriptive data). As expected, when comparing the experiments, we found that staying awake increased subjective sleepiness (sleep/wake × time point interaction: *F*_(3,123)_ = 46.41, *p* ≤ 0.001, η_p_^2^ = 0.53). Follow‐up comparisons verified that this effect focused on the morning and retrieval phase assessments in both conditions (all *p* ≤ 0.001). In experiment 2 (retention/wake), mefloquine induced a trend towards reduced subjective sleepiness (*t*_(11)_ = −1.83, *p* = 0.095, all other *p* ≥ 0.11) and improved reaction speed during retrieval (*t*_(10)_ = 2.30, *p* = 0.044, all other *p* ≥ 0.13). Across experiments, a condition × time‐point interaction (*F*_(3,38)_ = 8.79, *p* = 0.005, η_p_^2^ = 0.19) was mainly driven by increased reaction speed under mefloquine at retrieval. Reaction speed was generally reduced during retrieval in the wake compared to the sleep groups (sleep/wake × time point: *F*_(3,38)_ = 25.85, *p* ≤ 0.001, η_p_^2^ = 0.41).

Mood was negatively affected by sleep deprivation (sleep/wake × time point: *F*_(3,123)_ = 10.30, *p* ≤ 0.001, η_p_^2^ = 0.20). An apparent time‐dependent influence of mefloquine on mood (condition × time point: *F*_(3,123)_ = 6.29, *p* ≤ 0.001, η_p_^2^ = 0.13) could not be pinpointed to any specific direction and/or time point. Tiredness was increased by sleep deprivation at the expected time points (sleep/wake × time point: *F*_(3,123)_ = 45.76, *p* ≤ 0.001, η_p_^2^ = 0.53) and, in line with the above results, mefloquine reduced tiredness mainly in the retrieval phase (condition × time point: *F*_(3,123)_ = 3.38, *p* = 0.022, η_p_^2^ = 0.08). Similarly, sleep deprivation reduced calmness at the two later time points (sleep/wake × time point: *F*_(3,123)_ = 5.65, *p* = 0.002, η_p_^2^ = 0.12).

Analyses of blood samples confirmed that the timing of mefloquine administration in the three experiments worked as planned. Serum mefloquine concentrations were strongly increased in the mefloquine as compared to the placebo condition in experiments 1 (retention/sleep) and 2 (retention/wake) in the evening, in the morning and at retrieval (all *p* ≤ 0.001), as well as in experiment 3 at retrieval (*t*_(11)_ = 10.21, *p* ≤ 0.001, all other time points *p* ≥ 0.15; see Supplementary Table S1). We corrected all statistical analyses for the signs of mefloquine residuals in placebo sessions taking place after the respective mefloquine sessions (see 2.1.7).

### 3.6 Mefloquine Does Not Affect Hippocampal Sharp‐Wave/Ripple Activity in Rodents

**FIGURE 3:**
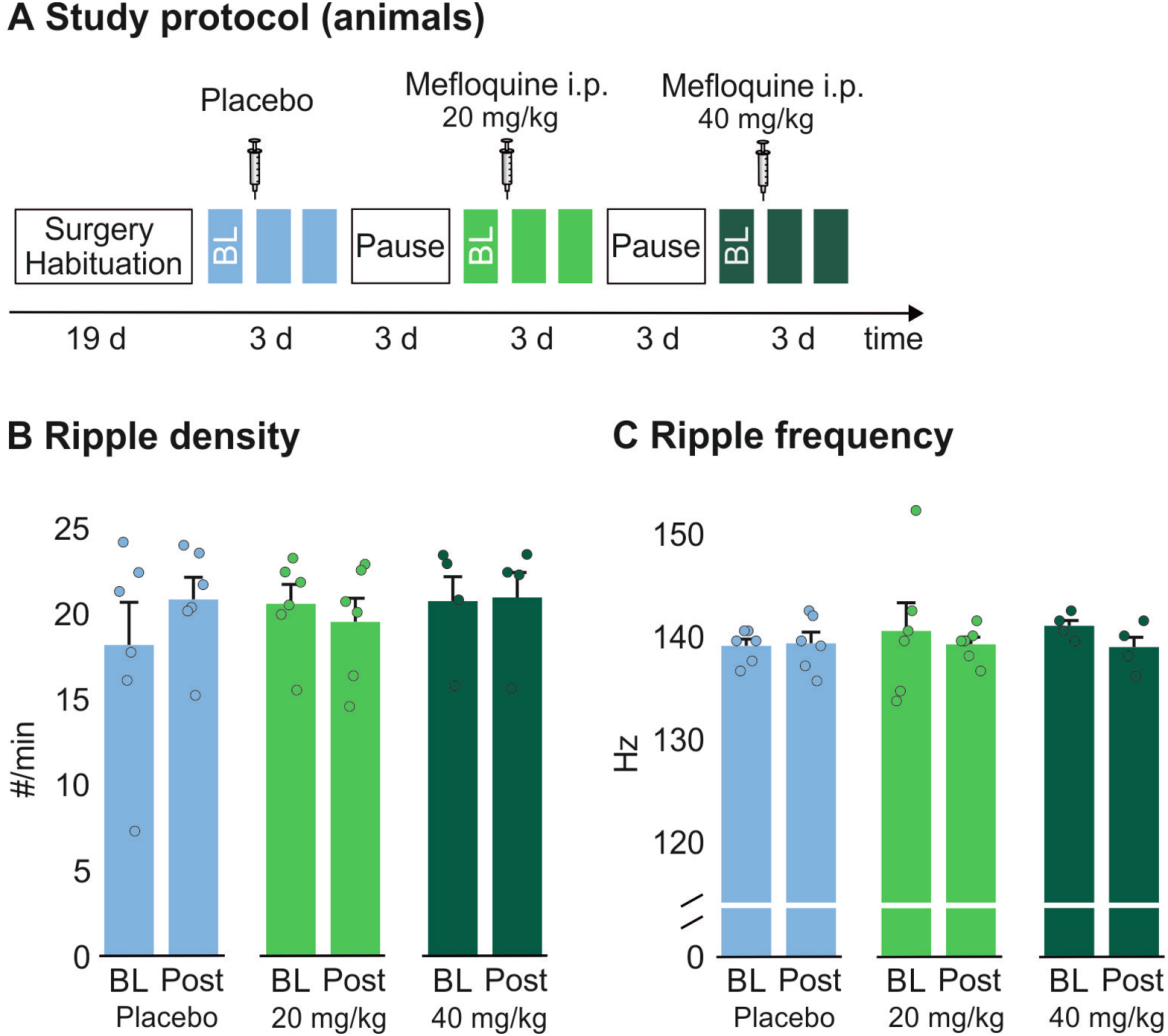
**Design and results of the experiment in rodents.** (A) After the implantation of electrodes for the recording of EEG, EMG and local field potentials (LFP) and subsequent recovery and habituation to the recording setting, we obtained 12‐h recordings (from 7:00 AM to 7:00 PM) once before (baseline, BL) and twice after i.p. placebo injection. After a pause of 3 days, the procedure was repeated with mefloquine instead of placebo at doses of 20 mg/kg and, 3 days later, 40 mg/kg body weight. (B) Density and (C) median frequency of hippocampal sharp‐wave/ripple complexes during baseline and averaged across the two postinjection recordings (post). *n* = 6 (placebo and 20 mg/kg dose) or *n* = 4 (40 mg/kg dose).

The additional experiments in rats were run to test our hypothesis that mefloquine attenuates hippocampal sharp‐wave/ripple activity. Recordings were obtained from 7:00 AM to 7:00 PM, that is, the animals' resting phase, before and for two consecutive days after the i.p. administration of placebo, 20 and 40 mg/kg of mefloquine; for each set of recordings, data of the two postadministration recordings were averaged (Figure 3A).

We could not confirm our main hypothesis. Analyses of hippocampal sharp wave/ripple density after mefloquine administration that included respective baseline values ruled out drug‐induced changes (Figure 3B; *F*_(2,6)_ = 0.13, *p* = 0.87 for drug × time; *p* > 0.16 for individual analyses of the 20 and 40 mg/kg doses). Median sharp wave/ripple frequency was likewise not systematically altered by mefloquine (Figure 3C; *F*_(2,4)_ = 1.36, *p* = 0.33). We moreover did not find mefloquine‐induced changes in time spent awake and the duration of SWS and REM sleep (all *p* > 0.19 for the interaction of drug × time, that is, placebo vs. mefloquine × before vs. after administration; see Supplementary Table S2 for descriptive data).

## 4 Discussion

In a series of experiments, we first administered mefloquine to block electrical coupling of neurons during nocturnal sleep in healthy humans and investigated its effect on sleep‐dependent and ‐independent memory retention by means of declarative word‐pair and two‐dimensional object location tasks as well as a procedural finger‐sequence tapping task. Partly confirming our hypotheses, mefloquine abolished the beneficial effect of sleep on the consolidation of memory for word pairs, whereas administering the drug before nocturnal wakefulness or retrieval testing did not affect declarative memory. Surprisingly, mefloquine improved retention of the procedural finger sequence tapping task across all three experiments. We did not find an effect of mefloquine on sleep architecture, but detected a marked mefloquine‐induced disruption of sleep spindle‐to‐slow oscillation coupling in humans. Our experiments in rodents did not indicate an attenuating effect of mefloquine on hippocampal sharp‐wave/ripple activity. This outcome is somewhat surprising considering in vitro findings in hippocampal slices from Cx36 knockout mice (Maier et al. 2002) but consistent with unchanged in vivo ripple activity in that mouse model (Buhl et al. 2003). We conclude that mefloquine disrupts mechanisms of consolidation that strengthen verbal declarative memory formation specifically during sleep, but promotes the formation of procedural memory independent of sleep. The effect of mefloquine on spindle‐to‐slow oscillation coupling, while sharp‐wave/ripple activity is not affected, suggests that electrical coupling is involved in extra‐hippocampal neuro‐electric processes of memory consolidation that contribute to rapid systems consolidation during sleep (Bontempi et al. 1999; Ji and Wilson 2007; Diekelmann and Born 2010; Brodt et al. 2023).

Our finding of reduced retention of word‐pair memory under mefloquine specifically during sleep identifies an as yet unknown mechanism of sleep‐dependent memory formation. This puts a new complexion on the ‘neuron doctrine,’ that is, the concept of the brain as a composite of discrete individual neurons that communicate exclusively via neurochemicals, which was first and foremost touted by Santiago Ramón y Cajal (Yuste 2015). This assumption was contested as early as the 1950s (Bullock 1959), and it is now clear that the direct electrical coupling of neurons via gap junctions indeed represents another mode of neuronal information exchange (Bennett and Zukin 2004; Söhl et al. 2005). Recent studies in rats indicate that gap junctions support acquisition and maintenance of fear memories (Bissiere et al. 2011). In those experiments, which did not manipulate sleep in the retention interval, i.p. and intrahippocampal administration of gap junction blockers (mefloquine and carbenoxolone) before or directly after learning blocked the subsequent retrieval of context fear memories, while the injection immediately preceding retrieval had no effect. Of note, blocking gap junctions during learning also inhibits hippocampal theta oscillations, which are known to support encoding (Fell and Axmacher 2011). In an observational study in humans (without placebo control), administering mefloquine to block electrical coupling impaired low‐level learning, that is, the acquisition of eye blink conditioning (Van Essen et al. 2010). Our study was not primarily designed to test encoding and employed complex memory tasks that recruit broader and different brain circuits, so that our results cannot be interpreted to refute the reports of mefloquine‐induced impairments in memory acquisition in rats (Bissiere et al. 2011) and humans (Van Essen et al. 2010).

Crucially, our findings in rats exclude a reduction in sharp‐wave/ripple activity in the hippocampus as a primary mechanism underlying the impairing effect of mefloquine on sleep‐dependent declarative memory formation. In contrast to the human participants, the rats were not trained on memory tasks before sleep recordings, which might have modulated the neurophysiological impact of gap junction blockade by mefloquine. Considering that hippocampal sharp‐wave/ripple activity is an intrinsic network property of hippocampal activity during NREM sleep (Buzsáki 2015), it is, however, reasonable to assume that relevant mefloquine effects would have emerged independent of preceding memory encoding. Administration of the human‐equivalent and twice the human‐equivalent dose of mefloquine ensured sufficient bioavailability of the compound, which was likely even enhanced compared to the human setting because of i.p. rather than oral administration. Therefore, we conclude that mefloquine may disrupt processes that support systems memory consolidation during sleep rather than exerting effects on hippocampal replay (Mölle and Born 2011; Staresina et al. 2015).

In the declarative domain, systems consolidation involves the transfer of information from hippocampus to neocortex (Dudai 2004). This process is assumed to bypass the plasticity‐stability dilemma, that is, to prevent interference that plagues systems wherein new information is encoded directly into the same networks that store old information (Marr 1971; Grossberg 1982, 1987). Sleep plays a crucial role for systems consolidation (Dudai et al. 2015), because the unique neurochemical milieu of SWS enables information flow from the hippocampus to the neocortex (Hasselmo 1999; Gais and Born 2004), which is orchestrated by neurophysiological sleep phenomena, that is, slow oscillations and sleep spindles (Mölle et al. 2002; Clemens et al. 2007; Mitra et al. 2016). Our finding that mefloquine reduced fast spindle band power (12–15 Hz) during the up‐state of the slow oscillation and in the slow spindle band (9–12 Hz) during the respective up to down‐state transition suggests that mefloquine impairs the neocortical‐hippocampal dialogue during sleep. Fast spindle density increases with the number of word pairs encoded before sleep (Gais et al. 2002), and boosting up‐state–associated fast spindle activity by auditory stimulation enhances word‐pair retention across sleep (Ngo et al. 2013). Rather than modulating the coupling between hippocampal ripples and spindle activity (Peyrache et al. 2011), blocking electrical coupling might have compromised the synchronization of spindling activity in thalamic reticular neurons, key generators of sleep spindles (Steriade et al. 1993). Indeed, in vitro and in vivo studies support this assumption (Landisman et al. 2002), and computational models predict a role for electric coupling in reticular neurons in the spread of low‐frequency activity (Fuentealba et al. 2004). Fast sleep spindles support plasticity in cortical neurons by increasing calcium influx (Sejnowski and Destexhe 2000; Niethard et al. 2018) and, thereby, systems consolidation. In this scenario, systems consolidation of word‐pair memory was disrupted by mefloquine eroding the fine‐tuned spindle‐slow oscillation complex. Our finding that memory retention tested with the object location task was unaffected by mefloquine supports this interpretation, because spatial information likely remains hippocampus‐dependent for a longer time (Winocur and Moscovitch 2011; Squire et al. 2015) and thus may be less affected by acute impairments of systems consolidation.

The conclusion that mefloquine affected processes of systems consolidation in our sleeping participants is buttressed by the results in the procedural memory domain, which further indicate that the detrimental memory effect of mefloquine is restricted to (nonspatial) declarative memory formation. Enhanced procedural retention due to mefloquine might at first glance be linked to its improving effect on vigilance, which may become behaviourally relevant after sleep deprivation. However, mefloquine did not improve performance on a control finger sequence learned at retrieval and generally enhanced performance accuracy for the retained, but not the control sequence, which makes this account unlikely. The enhancement of sensorimotor memory by mefloquine might rather point towards reduced hippocampal‐striatal competition, because such a reduction of competition of memory systems has been suggested to be the mechanism that supports procedural memory consolidation during postlearning sleep (Albouy et al. 2013). Mefloquine might induce a corresponding brain state of low competition independent of sleep. This assumption is in accordance with the role of Cx36 gap junctions for oscillatory activity in the striatum (Cummings et al. 2008), but also the inferior olivary nucleus (Van Der Giessen et al. 2008) and the cerebellar cortex (Vervaeke et al. 2010; Robinson et al. 2017). Mefloquine effects in these regions, as well as their interconnecting circuits, might in sum have contributed to the observed improvements in motor learning.

A main strength of our experimental approach is the combination of experiments in human participants and rats. The direct measurement of sharp‐waves/ripples in the hippocampus permits the mechanistic interpretation of behavioural effects and of scalp‐recorded electrophysiological changes in humans. Our findings in rats indicate that the electrical coupling of neurons in hippocampal networks is not essential for the sleep‐dependent formation of declarative memories, but that electrical synapses may rather support mechanisms that go beyond the hippocampal replay of memory traces. A shortcoming of our study is that similar to previous experiments (Buhl et al. 2003; Frisch et al. 2005; Bissiere et al. 2011), it relied on male‐only samples, so that potential sex differences could not be detected (Shansky and Murphy 2021). Another limitation concerns the unexpectedly weak retention of word‐pair memory in the placebo group of experiment 3, in which sleep during the retention interval did not benefit verbal declarative memory as strongly as in experiment 1, thereby potentially masking negative mefloquine effects on retrieval. Still, the absence of mefloquine effects on verbal declarative memory retained across nocturnal wakefulness corroborates that the drug acted on sleep‐dependent mechanisms of memory retention. Finally, the exclusive use of mefloquine to block electrical synapses was imposed by ethical considerations because we aimed to restrict side effects that might have been induced by using alternative (or additional) pharmacological agents (Juszczak and Swiergiel 2009). While mefloquine is a potent blocker of Cx36, the primary gap junction protein in electrical synapses of the mammalian CNS, it is important to keep in mind that it also exerts effects that are unrelated to gap junctions, for example, on acetylcholinesterase activity, adenosine receptors and ATP‐sensitive K + channels (Lim and Go 1985; Cruikshank et al. 2004; Manjarrez‐Marmolejo and Franco‐Pérez 2016). However, mefloquine induces less pronounced anticholinesterase effects than other antimalarials, and we did not observe the effects on REM sleep that are usually elicited by more potent cholinesterase inhibitors (Sitaram et al. 1976; Hasselmo 1999; Nissen et al. 2006). Moreover, IC_50_ values of mefloquine, which reflect its potency to inhibit biological processes (lower values indicate higher potency), are much smaller for Cx36 (0.3 μM) than for ATP‐sensitive K + channels (3 μM) or, for example, L‐type Ca channels (10 μM), suggesting that in the present experiments, mefloquine primarily acted on Cx36 (Gribble et al. 2000; Van Essen et al. 2010), although contributions of mechanisms unrelated to electrical synapses cannot be completely excluded. In this context it is notable that mefloquine's effect on fear learning in rats is identical to those of the general gap‐junction blocker carbonoxolone, which has a different spectrum of nongap junction‐related effects (Bissiere et al. 2011), suggesting that the memory impact of both compounds is mediated via gap junctions.

## 5 Conclusion

We demonstrate that administering mefloquine to block electrical coupling during nocturnal sleep selectively impairs declarative word‐pair retention in humans; based on our findings in rodents, this effect is not mediated via inhibition of hippocampal sharp‐wave/ripples (Girardeau et al. 2009; van de Ven et al. 2016) but might rather stem from impaired oscillatory coordination between sleep spindles and slow oscillations compromising hippocampus‐to‐neocortex memory transfer. Our data indicate a functional role for electrical synapses in the sleep‐dependent consolidation of declarative memory. Concurrent sleep and declarative memory dysfunction is a common feature of cognitive impairments that hallmark ailments such as schizophrenia (Manoach et al. 2016) and Alzheimer's disease (Lim et al. 2013), but also emerge during normal aging (Mander et al. 2013; Helfrich et al. 2018). Considering that electrical synapses have been suggested to play a role both in schizophrenia (Aleksic et al. 2007) and Alzheimer's disease (Giaume et al. 2019), unravelling their role in sleep‐dependent memory formation in humans may open new therapeutic avenues (MacEwan et al. 2016; Feld and Born 2020).

## Author Contributions

**Gordon B. Feld:** Conceptualization, funding acquisition, formal analysis, methodology, supervision, validation, visualization, writing original draft. **Niels Niethard:** Conceptualization, formal analysis, methodology, validation, visualization, writing review edition. **Jianfeng Liu:** Investigation, formal analysis, visualization. **Sandra Gebhardt:** Investigation. **Lisa Kleist:** Investigation. **Kerstin Brugger:** Investigation. **Andreas Fritsche:** Supervision. **Jan Born:** Conceptualization, funding acquisition, resources. **Hong‐Viet V. Ngo:**Formal analysis. **Manfred Hallschmid:** Conceptualization, funding acquisition, methodology, project administration, resources, supervision, validation, writing original draft, writing review edition.

## Funding

This work was supported by Gemeinnützige Hertie‐Stiftung, Bundesministerium für Bildung und Forschung (01GI0925) and Deutsche Forschungsgemeinschaft (SFB 654 and FE 1617/2‐1).

## Ethics Statement

The experiments in humans and, respectively, animals were approved by the Ethics Committee of the Medical Faculty at the University of Tübingen and the Baden‐Württemberg state authority.

## Conflicts of Interest

The authors declare no conflicts of interest.

## Supporting information


**Figure S1:** **Sleep results of experiment 1.** Time‐frequency plots time‐locked to the down‐state of the slow oscillation during SWS at Fz, Cz and Pz. Effects of mefloquine on the coupling of sleep spindles to slow oscillations (0.75 Hz) were analysed by identifying the peaks of the slow oscillation down‐states and calculating EEG power between 5 and 20 Hz in 3‐s time windows centered on the down‐states (x‐axes: ‘0’). Upper and midline panels depict this time‐frequency representation for the placebo and, respectively, the mefloquine conditions. Bottom panels depict the differences between the two conditions, with the areas of statistically significant differences outlined in black (two‐tailed paired‐samples *t* tests corrected for multiple comparisons). Colour bars to the right of the panels indicate scales of spectral power relative to the average power obtained between −1.5 and −1.4 s with respect to the slow oscillation down‐state (upper and midline panels) and of respective differences between conditions (lower panels). Note that the Pz plots are also depicted in **Figure 2B** and shown here for comparison with Fz and Cz.
**Table S1:** Mean (SEM) serum mefloquine concentrations (ng/ml), sleepiness (Stanford Sleepiness Scale), reaction speed (psychomotor vigilance task), mood (multidimensional mood questionnaire) and word generation (verbal fluency test).
**Table S2:** Mean (± SEM) duration of sleep stages in the rodent experiment.

## Data Availability

The data supporting this study's findings are available from the corresponding author upon reasonable request.
