# Supplementary material for: Electrical Synapses Contribute to Sleep‐Dependent Declarative Memory Retention
Source: Eur J Neurosci. 2026 Jan 23;63(2):e70401. doi: 10.1111/ejn.70401 (PMC12828869; doi:10.1111/ejn.70401)
Supplement: Supplementary file 1 — Figure S1: Sleep results of experiment 1. Time‐frequency plots time‐locked to the down‐state of the slow oscillation during SWS at Fz, Cz and Pz. Effects of mefloquine on the coupling of sleep spindles to slow oscillations (0.75 Hz) were analysed by identifying the peaks of the slow oscillation down‐states and calculating EEG power between 5 and 20 Hz in 3‐s time windows centered on the down‐states (x‐axes: ‘0’). Upper and midline panels depict this time‐frequency representation for the placebo and, respectively, the mefloquine conditions. Bottom panels depict the differences between the two conditions, with the areas of statistically significant differences outlined in black (two‐tailed paired‐samples t tests corrected for multiple comparisons). Colour bars to the right of the panels indicate scales of spectral power relative to the average power obtained between −1.5 and −1.4 s with respect to the slow oscillation down‐state (upper and midline panels) and of respective differences between conditions (lower panels). Note that the Pz plots are also depicted in Figure 2B and shown here for comparison with Fz and Cz. Table S1: Mean (SEM) serum mefloquine concentrations (ng/ml), sleepiness (Stanford Sleepiness Scale), reaction speed (psychomotor vigilance task), mood (multidimensional mood questionnaire) and word generation (verbal fluency test). Table S2: Mean (± SEM) duration of sleep stages in the rodent experiment. [file EJN-63-0-s001.docx]

Feld et al.:

**Electrical synapses contribute to sleep-dependent
declarative memory retention**

*Supporting information*

**
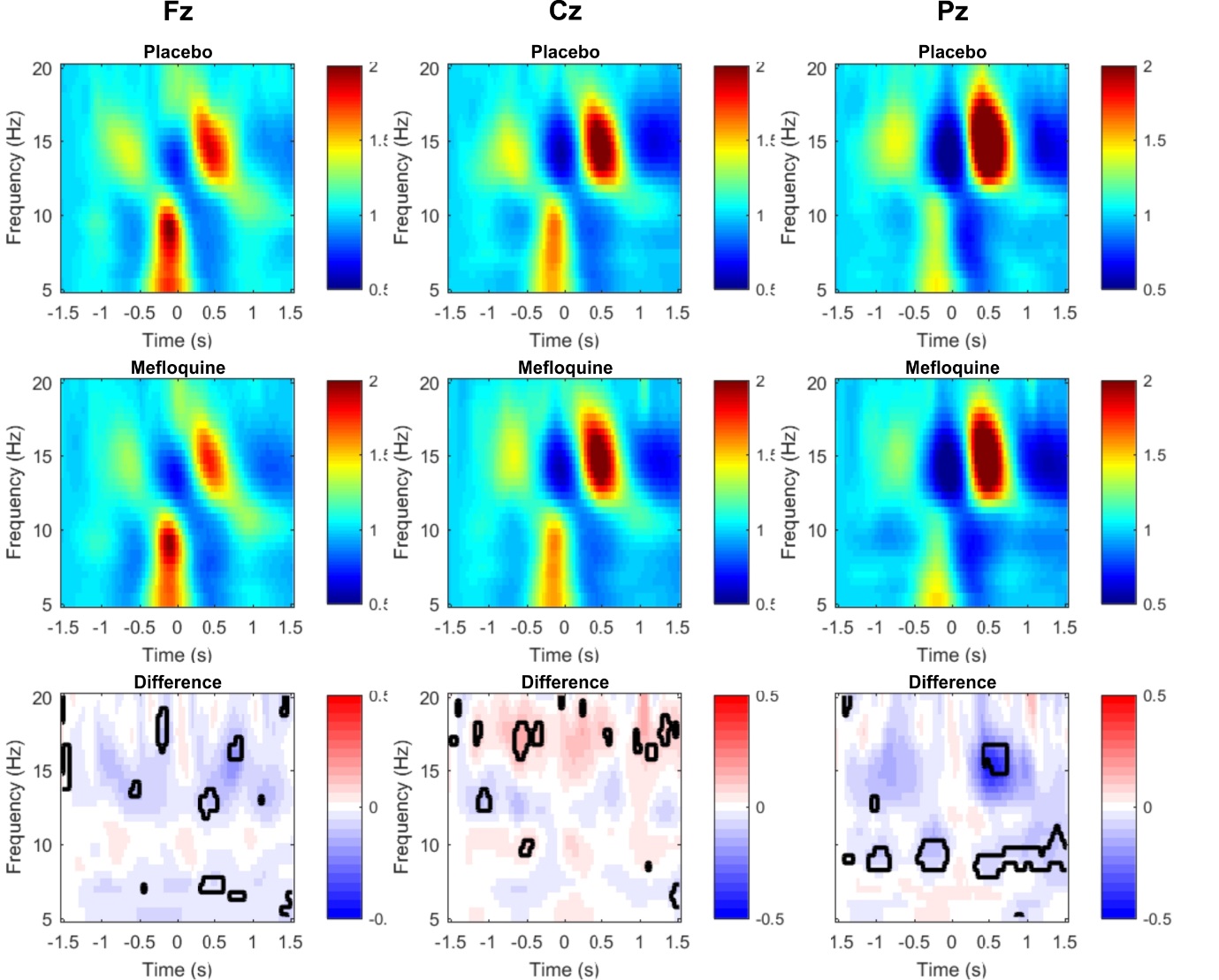
**

**Supplementary Figure S1. Sleep results of experiment 1.** Time-frequency plots time-locked to the down-state of the slow oscillation during SWS at Fz, Cz and Pz. Effects of mefloquine on the coupling of sleep spindles to slow oscillations (0.75 Hz) were analyzed by identifying the peaks of the slow oscillation down-states and calculating EEG power between 5 and 20 Hz in 3-second time windows centered on the down-states (x-axes: ‘0’). Upper and midline panels depict this time-frequency representation for the placebo and, respectively, the mefloquine conditions. Bottom panels depict the differences between the two conditions, with the areas of statistically significant differences outlined in black (two-tailed paired-samples t-tests corrected for multiple comparisons). Colour bars to the right of the panels indicate scales of spectral power relative to the average power obtained between -1.5 and -1.4 s with respect to the slow oscillation down-state (upper and midline panels) and of respective differences between conditions (lower panels). Note that the Pz plots are also depicted in **Figure 2B** and shown here for comparison with Fz and Cz.

**Supplementary Table S1:** Mean (SEM) serum mefloquine concentrations (ng/ml), sleepiness (Stanford Sleepiness Scale), reaction speed (psychomotor vigilance task), mood (multi-dimensional mood questionaire) and word generation (verbal fluency test).

|  | **Experiment 1 (Retention/sleep)** | | | | **Experiment 2 (Retention/wake)** | | | | **Experiment 3 (Retrieval)** | | | |
| --- | --- | --- | --- | --- | --- | --- | --- | --- | --- | --- | --- | --- |
|  | Mefloquine | | Placebo | | Mefloquine | | Placebo | | Mefloquine | | Placebo | |
| **Mefloquine levels** | |  |  |  |  |  |  |  |  |  |  |  |
| Evening | 188.95 | (18.59) | 13.68 | (5.31) | 285.00 | (38.95) | 16.08 | (9.28) | 0.00 | NA | 13.17 | (8.56) |
| Morning | 201.84 | (10.81) | 12.89 | (5.02) | 332.50 | (30.17) | 10.42 | (7.08) | 0.00 | NA | 12.00 | (7.73) |
| Retrieval phase | 207.11 | (12.90) | 13.16 | (5.18) | 312.27 | (29.22) | 11.67 | (8.08) | 354.17 | (32.28) | 11.92 | (7.40) |
| **Sleepiness** |  |  |  |  |  |  |  |  |  |  |  |  |
| Learning phase | 2.58 | (0.25) | 2.68 | (0.28) | 3.00 | (0.43) | 2.50 | (0.26) | 2.46 | (0.24) | 2.83 | (0.27) |
| Evening | 4.26 | (0.29) | 4.32 | (0.32) | 3.58 | (0.45) | 3.25 | (0.39) | 4.21 | (0.29) | 4.50 | (0.38) |
| Morning | 2.68 | (0.25) | 2.84 | (0.24) | 5.42 | (0.43) | 5.42 | (0.36) | 3.00 | (0.3) | 3.33 | (0.26) |
| Retrieval phase | 1.84 | (0.23) | 2.16 | (0.22) | 4.67 | (0.40) | 5.42 | (0.36) | 1.67 | (0.14) | 1.67 | (0.14) |
| **Reaction speed** |  |  |  |  |  |  |  |  |  |  |  |  |
| Learning phase | 3.68 | (0.08) | 3.60 | (0.11) | 3.68 | (0.08) | 3.74 | (0.09) | 3.53 | (0.13) | 3.63 | (0.11) |
| Retrieval phase | 3.78 | (0.09) | 3.69 | (0.10) | 3.58 | (0.13) | 3.40 | (0.15) | 3.71 | (0.16) | 3.63 | (0.13) |
| **Mood** |  |  |  |  |  |  |  |  |  |  |  |  |
| *Positive mood* |  |  |  |  |  |  |  |  |  |  |  |  |
| Learning phase | 4.45 | (0.07) | 4.33 | (0.06) | 4.19 | (0.16) | 4.50 | (0.11) | 4.21 | (0.18) | 4.44 | (0.08) |
| Evening | 4.47 | (0.08) | 4.38 | (0.12) | 4.02 | (0.24) | 4.21 | (0.11) | 4.31 | (0.10) | 4.35 | (0.10) |
| Morning | 4.33 | (0.14) | 4.45 | (0.10) | 3.27 | (0.2) | 3.71 | (0.20) | 3.92 | (0.29) | 4.23 | (0.08) |
| Retrieval phase | 4.54 | (0.15) | 4.49 | (0.12) | 3.85 | (0.24) | 3.48 | (0.31) | 4.65 | (0.06) | 4.58 | (0.09) |
| *Tiredness (inv.)* |  |  |  |  |  |  |  |  |  |  |  |  |
| Learning phase | 3.62 | (0.19) | 3.45 | (0.22) | 3.38 | (0.30) | 3.96 | (0.18) | 3.48 | (0.19) | 3.65 | (0.15) |
| Evening | 2.80 | (0.19) | 2.59 | (0.21) | 3.15 | (0.30) | 3.42 | (0.24) | 2.94 | (0.17) | 2.90 | (0.19) |
| Morning | 3.57 | (0.19) | 3.59 | (0.17) | 2.08 | (0.24) | 2.02 | (0.16) | 3.33 | (0.19) | 3.31 | (0.11) |
| Retrieval phase | 3.97 | (0.17) | 3.67 | (0.21) | 2.40 | (0.27) | 1.94 | (0.23) | 4.19 | (0.15) | 4.17 | (0.16) |
| *Calmness* |  |  |  |  |  |  |  |  |  |  |  |  |
| Learning phase | 4.21 | (0.13) | 4.13 | (0.14) | 3.94 | (0.13) | 4.40 | (0.13) | 3.94 | (0.29) | 4.08 | (0.17) |
| Evening | 4.22 | (0.11) | 4.03 | (0.18) | 3.90 | (0.2) | 4.25 | (0.16) | 4.25 | (0.15) | 4.38 | (0.09) |
| Morning | 4.21 | (0.13) | 4.08 | (0.11) | 3.44 | (0.17) | 3.79 | (0.15) | 3.75 | (0.23) | 4.27 | (0.10) |
| Retrieval phase | 4.11 | (0.17) | 4.04 | (0.21) | 3.63 | (0.18) | 3.54 | (0.23) | 4.31 | (0.15) | 4.33 | (0.17) |
| **Word generation** |  |  |  |  |  |  |  |  |  |  |  |  |
| Letter | 16.16 | (1.05) | 17.37 | (1.16) | 14.92 | (1.41) | 13.92 | (1.02) | 14.58 | (1.58) | 14.25 | (1.30) |
| Category | 20.42 | (0.84) | 21.84 | (0.98) | 17.83 | (1.30) | 15.58 | (1.25) | 19.58 | (0.95) | 17.67 | (1.30) |
| Total | 36.58 | (1.64) | 39.21 | (1.80) | 32.75 | (2.45) | 29.50 | (1.86) | 34.17 | (2.10) | 31.92 | (2.17) |

**Supplementary Table S2:** Mean (± SEM) duration of sleep stages in the rodent experiment.

|  | **Placebo** | | **20 mg/kg** | | **40 mg/kg** | |
| --- | --- | --- | --- | --- | --- | --- |
|  | Baseline | Post-injection | Baseline | Post-injection | Baseline | Post-injection |
| **Wake** | 253.94 ± 18.19 | 266.82 ± 11.78 | 329.19 ± 34.75 | 330.21 ± 25.34 | 359.08 ± 31.26 | 323.42 ± 16.61 |
| **SWS** | 316.64 ± 17.60 | 303.10 ± 10.16 | 271.75 ± 29.84 | 262.65 ± 21.480 | 252.13 ± 24.26 | 279.67 ± 11.23 |
| **REM** | 76.61 ± 4.630 | 73.01 ± 4.13 | 53.67 ± 6.21 | 46.94 ± 6.53 | 32.46 ± 7.45 | 39.58 ± 4.50 |

Twelve-hour baseline recordings (from 7:00 am to 7:00 pm) were obtained once before and twice after i.p. injection of placebo, of 20 mg/kg and of 40 mg/kg mefloquine. Duration (min) of wakefulness, SWS and REM sleep at baseline and averaged across the two post-injection recordings are shown. Overall statistical analyses did not indicate significant treatment effects (all p>0.19 for drug × time); n=6 (placebo and 20 mg/kg) and n=4 (40 mg/kg).
